# Human growth hormone inclusion bodies present native-like secondary and tertiary structures which can be preserved by mild solubilization for refolding

**DOI:** 10.1186/s12934-022-01887-1

**Published:** 2022-08-17

**Authors:** Rosa Maria Chura-Chambi, Chuck Shaker Farah, Ligia Morganti

**Affiliations:** 1 Centro de Biotecnologia, Instituto de Pesquisas Energéticas e Nucleares, IPEN-CNEN/SP, São Paulo, SP Brazil; 2 Departamento de Bioquímica, Instituto de Química da Universidade de São Paulo, São Paulo, SP Brazil

**Keywords:** Protein refolding, Inclusion bodies, Human growth hormone, High hydrostatic pressure, Alkaline pH

## Abstract

**Background:**

Native-like secondary structures and biological activity have been described for proteins in inclusion bodies (IBs). Tertiary structure analysis, however, is hampered due to the necessity of mild solubilization conditions. Denaturing reagents used for IBs solubilization generally lead to the loss of these structures and to consequent reaggregation due to intermolecular interactions among exposed hydrophobic domains after removal of the solubilization reagent. The use of mild, non-denaturing solubilization processes that maintain existing structures could allow tertiary structure analysis and increase the efficiency of refolding.

**Results:**

In this study we use a variety of biophysical methods to analyze protein structure in human growth hormone IBs (hGH-IBs). hGH-IBs present native-like secondary and tertiary structures, as shown by far and near-UV CD analysis. hGH-IBs present similar λ_max_ intrinsic Trp fluorescence to the native protein (334 nm), indicative of a native-like tertiary structure. Similar fluorescence behavior was also obtained for hGH solubilized from IBs and native hGH at pH 10.0 and 2.5 kbar and after decompression. hGH-IBs expressed in *E. coli* were extracted to high yield and purity (95%) and solubilized using non-denaturing conditions [2.4 kbar, 0.25 M arginine (pH 10), 10 mM DTT]. After decompression, the protein was incubated at pH 7.4 in the presence of the glutathione-oxidized glutathione (GSH-GSSG) pair which led to intramolecular disulfide bond formation and refolded hGH (81% yield).

**Conclusions:**

We have shown that hGH-IBs present native-like secondary and tertiary structures and that non-denaturing methods that aim to preserve them can lead to high yields of refolded protein. It is likely that the refolding process described can be extended to different proteins and may be particularly useful to reduce the pH required for alkaline solubilization.

## Background

*Escherichia coli* is an extremely efficient host for the production of recombinant proteins that do not require glycosylation. Depending on protein characteristics and culture conditions, genetically engineered bacteria can express soluble proteins or accumulate insoluble deposits in the cytoplasm known as inclusion bodies (IBs) [1]. A major advantage of obtaining proteins as IBs is the facility of purification of insoluble aggregates, which often eliminates the need for downstream purification. The disadvantage is the need to develop an efficient refolding process to obtain functional molecules with the native conformation.

The analysis of secondary structure of insoluble IBs, generally performed by FT-IR, has revealed the presence of native-like secondary structure [2]. Biological activity has also been reported in some cases [3]. It is, therefore, advisable to attempt to solubilize IBs under mild conditions that maintain the existing structures during refolding, as opposed to traditional refolding techniques that use high concentrations of urea and guanidine which promote complete protein denaturation and frequently lead to reaggregation. Solubilization techniques described as mild include the use of organic solvents in combination with 2 to 3 M urea [4, 5], low urea concentrations [6], high pressures in combination with guanidine hydrochloride [7–11] and alkaline pH (≥ 12) in combination with 2 M urea [12–14]. Utilization of detergents have also been reported [15]. However, even these processes that are described as mild frequently can lead to tertiary structure loss.

High hydrostatic pressures promote solubilization of aggregates by weakening hydrophobic and electrostatic interactions [16]. Protein secondary structures are relatively insensitive to high pressure and may even be stabilized at pressures below 5 kbar [17, 18].

Proteins become negatively charged at a pH above their isoelectric point, which increases electrostatic repulsion between protein molecules and hydration of charged residues. As a result, solubility is increased. However, exposure to extremely alkaline pH conditions may cause irreversible chemical modifications and/or denaturation. Therefore, although solubilization of IBs using alkaline pH may be attractive for its simplicity and low cost, it may not be applicable to proteins that are especially sensitive to extreme pH [19, 20].

We have recently described the combination of high pressure and alkaline pH for IBs solubilization and subsequent refolding [21–23]. The fact that the pH required for the solubilization of proteins in IBs is reduced when this condition is associated with high pressure can alleviate the problems that arise from the use of extremely alkaline pH.

Human growth hormone (hGH) is a 22-kDa monomeric protein with a core formed by a four-helix bundle that contains two intramolecular disulfide bonds C53–C165 and C182–C189 [24]. In the present study, we demonstrate that hGH-IBs present native-like secondary and tertiary structures. The association of two complementary and non-denaturing tools, high pressure and alkaline pH, for solubilization of proteins in IBs and refolding was also analyzed. We show that high pressure in association with pH 10.0 in the presence of reducing reagent and arginine completely solubilizes hGH-IBs with a very low degree of protein denaturation, enabling high-yield refolding. In addition, oxidative folding conditions, which promotes the formation and rearrangement of protein disulfide bonds (S–S-bonds) aiming at the formation of native disulfide bridges, are described. The technique we describe is an extremely attractive protein refolding protocol with widespread applicability.

## Methods

### Expression of hGH

A codon-optimized hGH gene was synthesized and cloned into the pET29 expression vector by Biomatik (Canada). *Escherichia coli* BL21 (DE3) (Novagen, USA) was used as host for recombinant protein expression. Bacteria were grown at 37 °C in 500 mL of the rich medium HKSII [25] in two 1L Erlenmeyer flasks under agitation. Induction of hGH expression was performed by addition of IPTG to 0.5 mM when culture reached optical density at 600 nm between 2.5 and 3.0 and cultivation continued at 30 °C for 16 h.

### hGH-IB isolation and purification

hGH-IBs isolation was performed as previously described [23]. Briefly, the culture was centrifuged at 3000×*g* for 15 min at 4 °C. The pellet was resuspended in 50 mL 0.1 M TrisHCl pH 7.4 + 5 mM EDTA. Lysozyme was added to reach a concentration of 50 µg/mL and the suspension was incubated for 15 min at room temperature. Sodium deoxycholate was added to 0.1% and bacterial lysis was performed by sonicating the suspension in ice until viscosity was lost. The suspension was centrifuged at 15,000×*g* for 15 min at 4 °C; the supernatant was discarded and the pellet was suspended in 50 mL of 0.1 M TrisHCl pH 7.4 containing 0.1% sodium deoxycholate and 5 mM EDTA and quickly sonicated to completely disperse the lumps. The suspension was centrifuged, and hGH-IBs were washed again. The pellet was suspended in 50 mL 50 mM TrisHCl buffer at pH 7.4 containing 1 mM EDTA, centrifuged and suspended in 20 mL of the same buffer.

### Polyacrylamide gel electrophoresis (SDS-PAGE)

SDS-PAGE was performed using 12% gel containing sodium dodecyl sulfate, stained with Coomassie Blue G-250. For protein analysis under reducing condition, the sample buffer also contained 0.1 M dithiothreitol (DTT).

### Quantification of hGH

For quantification of the protein in hGH-IBs, suspensions were solubilized by dilution in 6 M GdnHCl and the absorbance at 280 nm was monitored. We used Abs 0.1% = 0.794 and and ε = 17,670 for determination of hGH concentration. Determination of the percentage of hGH monomer, dimer and higher molecular isoforms of soluble hGH was performed by HPSEC. Analysis of SDS-PAGE bands using Image J program was performed for determination of the percentage of refolding of hGH in comparison to hGH-IBs and also for the determination of the percentage of contaminants.

### High hydrostatic pressure

The buffers used for the solubilization tests in different pH were 50 mM TrisHCl for pH 7.0 to 9.0 and 50-mM CAPS for pH 10.0 to 12.0 in the presence of 1 mM EDTA. A volume of 1 mL hGH-IBs suspensions containing 1 mg protein were introduced into plastic bags that were sealed and placed into a larger plastic bag that was vacuum-sealed. The bag was put into a pressure vessel (R4-6-40, High Pressure Equipment, USA) that was pressurized to 2.4 kbar using oil as a transmission fluid using a suitable high-pressure pump (PS-50, High Pressure Equipment, USA) at 20 °C. Compression was carried out for 90 min and the decompression was accomplished directly to 1 bar. The pH of the arginine solutions was adjusted to the desired pH, because this amino acid confers alkaline pH (approximately pH 11.0) to solutions. For oxidative folding, hGH at a concentration of 1 mg/mL in CAPS 50 mM pH 10.0, 1 mM EDTA, 10 mM DTT and 0.25 M Arg that had been adjusted to pH 10.0 was solubilized at 2.4 kbar for 90 min and centrifuged to remove remaining insoluble material. The pH of the supernatant solution was diminished by tenfold dilution in TrisHCl 50 mM pH 7.4 in the presence of GSSG at the concentration of 2 mM, incubated for 48 h at 4 °C, dialyzed overnight against TrisHCl 25 mM pH 7.4 and centrifuged to remove insoluble aggregates. We assumed that 1 mol of DTT completely reacts with 1 mol of GSSG to create 2 mol of GSH. As example, the final concentrations of glutathiones in a sample of hGH-IBs solubilized in the presence of 10 mM DTT and diluted tenfold in TrisHCl and 2 mM GSSG were 2 mM GSH and 1 mM GSSG.

### Fluorescence and light scattering (LS)

Cary Eclipse spectrofluorimeter (Varian) was used for Fluorescence and LS determinations. Data were collected using 1 cm optical path cuvettes, and the measures were accomplished at a 90° angle relative to the incident light using a 1 s response time and reading speed of 240 nm/minute. For intrinsic fluorescence we used an excitation at 280 nm and fluorescence intensity was determined between 300 and 400 nm. LS measurements were performed with excitation at 320 nm, and emission collected from 315 to 325 nm.

For studies under pressure, a high-pressure cell equipped with 3 optical sapphire windows (ISS, USA), was connected to a pressure generator (High pressure equipment, USA) and placed inside the spetrofluorimeter. Water was utilized as pressure-transmitting fluid. Round quartz cuvettes filled with the samples and sealed with flexible polyethylene caps were placed into the high-pressure cell and subjected to high hydrostatic pressure and fluorescence and LS were determined.

### Circular dichroism

Far and near-UV CD spectra were recorded using a Jasco-720 spetro-polarimeter in the wavelength ranges of 260–190 nm and 330–250 nm respectively at 21 °C. Monomeric refolded hGH and hGH solubilized from hGH-IBs at pH 10.0 were analyzed in a 2 mm path length cuvette at the molarity of 12 µM for far-UV and in a 1 cm cuvette and 40 µM for near-UV. Three independent spectra were acquired for each sample.

### High performance size exclusion chromatography (HPSEC)

For HPSEC, a G2000 SW column (60 cm–7.5 mm i.d., particle size 10 μm, pore size 125 Å) (Tosoh Bioscience, USA) coupled to a 7.5 cm–7.5 mm i.d. SW guard column was used. The mobile phase was 0.025 M ammonium bicarbonate, pH 7.0 at a flow rate of 1.0 mL/min. The sample elution was detected by UV absorbance at a wavelength of 220 nm. Spectra of purified hGH, without initial methionine, was used as a control of time of retention and for quantification.

### NMR of ^15^N-hGH

hGH-IBs was expressed in *E. coli* cultivated in M9 medium with ^15^N ammonium chloride. The washed IBs were solubilized at a concentration of 2 mg hGH/mL at 2.4 kbar and pH 10.0 in the presence of 0.25 M Arg and 10 mM DTT. The protein was then diluted tenfold in Tris HCl 50 mM containing 2 mM GSSG and incubated for 48 h at 4 °C for refolding. The protein was then dialyzed against Tris HCl 25 mM pH 7.4 and concentrated. The monomers were separated from the higher molecular weight isoforms by HPSEC and concentrated again to a concentration of 109 µM. NMR spectra were recorded at 298.15 K on a Bruker Advance III 600 MHz spectrometer operating at a magnetic field of 14.09 T and utilizing an inverse TBI probe with 5 mm.

## Results

### Effect of pH and high pressure on solubilization of hGH-IBs

**Fig. 1:**
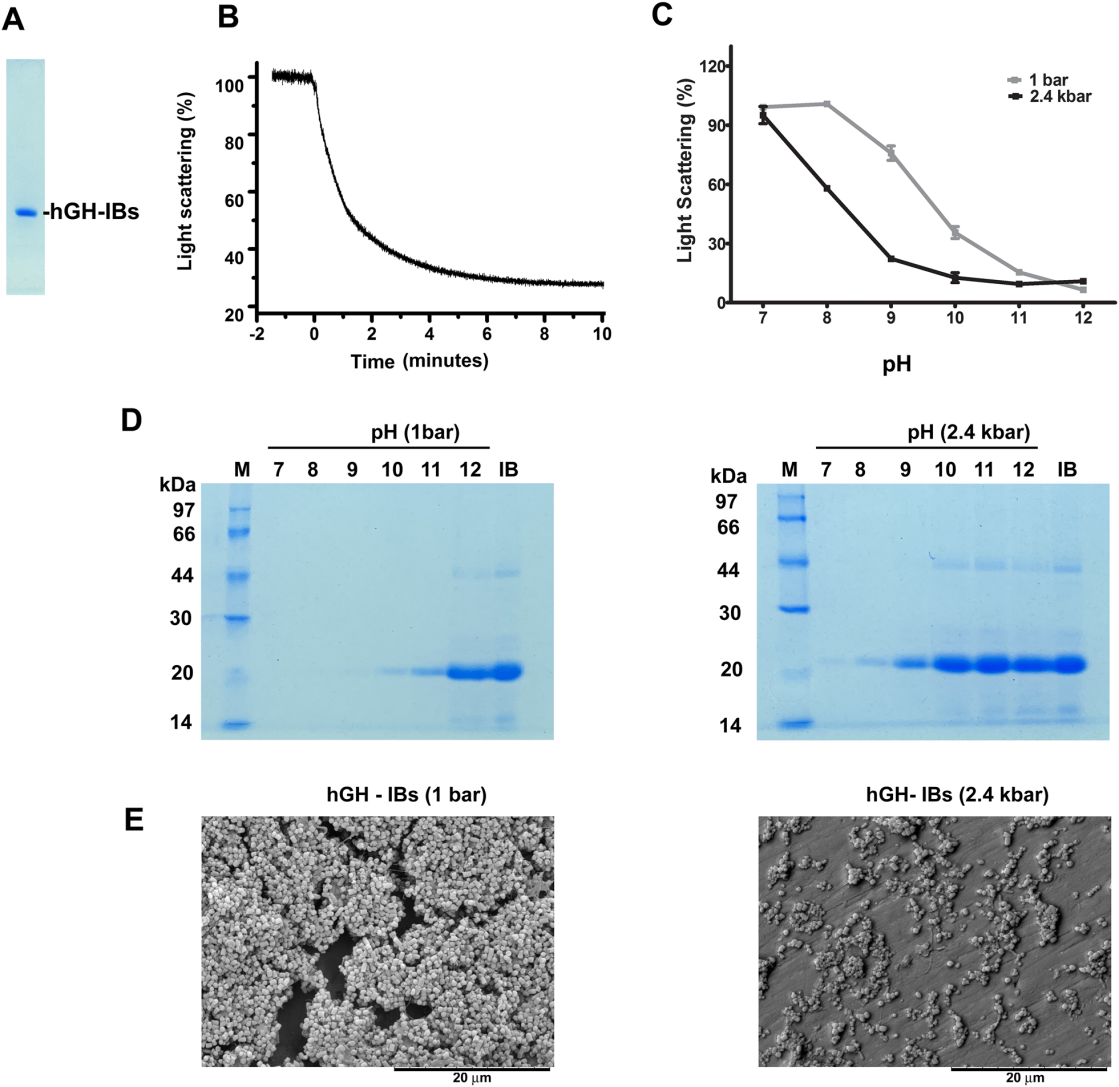
Alkaline pH and high pressure promote solubilization of hGH-IBs. **A** SDS-PAGE of hGH-IBs (reducing condition); **B** real-time determination of LS of a suspension of hGH-IBs subjected to 2.4 kbar at pH 10.0; **C** determination of LS of suspensions of hGH-IBs that had been subjected to 2.4 kbar or maintained at atmospheric pressure for 90 min at 20 °C. This experiment was performed in duplicate (1 bar) or triplicate (2.4 kbar); **D** SDS-PAGE of supernatants of samples subjected to 1 bar or to 2.4 kbar (reducing conditions); **E** scanning electron micrography of hGH-IBs and of the remaining insoluble fraction of a sample that was subjected to 2.4 kbar for 90 min at pH 9.0

An average of 700 ± 104 mg hGH was obtained in the washed hGH-IBs from 1 L induced *E. coli* culture. The purity of hGH in IBs is very high: 95% (Fig. 1A), which can be assigned to two factors: the high levels of expression and the washings of the insoluble aggregates, that reduce the presence of contaminants.

We have previously shown that the association of high pressure and alkaline pH is effective to solubilize IBs [21]. The solubilization of hGH-IBs at pH 10.0 and 2.4 kbar takes less than 10 min, as verified by a drop in light scattering (LS) during compression (Fig. 1B). High pressure enhances solubilization of hGH-IBs, as shown by lower values of visible LS obtained for the suspensions subjected to 2.4 kbar for 90 min than samples that were maintained during equal period of time at 1 bar (Fig. 1C) and, as expected, IBs become more soluble as the pH increases. Soluble hGH is obtained in the supernatant of the hGH-IBs incubated at 2.4 kbar at a pH as low as 9.0 and it is almost completely solubilized at pH 10.0 and higher. The aggregates that were incubated at 1 bar are completely solubilized only at pH 12.0 (Fig. 1D).

Analysis of hGH-IBs by scanning electron microscopy shows the characteristic structures presenting a cylindrical format with approximately 1.0 ± 0.3 µM length × 0.6 ± 0.1 µM width (Fig. 1E). As expected, the amount of hGH-IBs was drastically reduced by application of 2.4 kbar at pH 9.0.

### Solubilization of hGH-IBs, dissociation of oligomers and oxidative folding

**Fig. 2:**
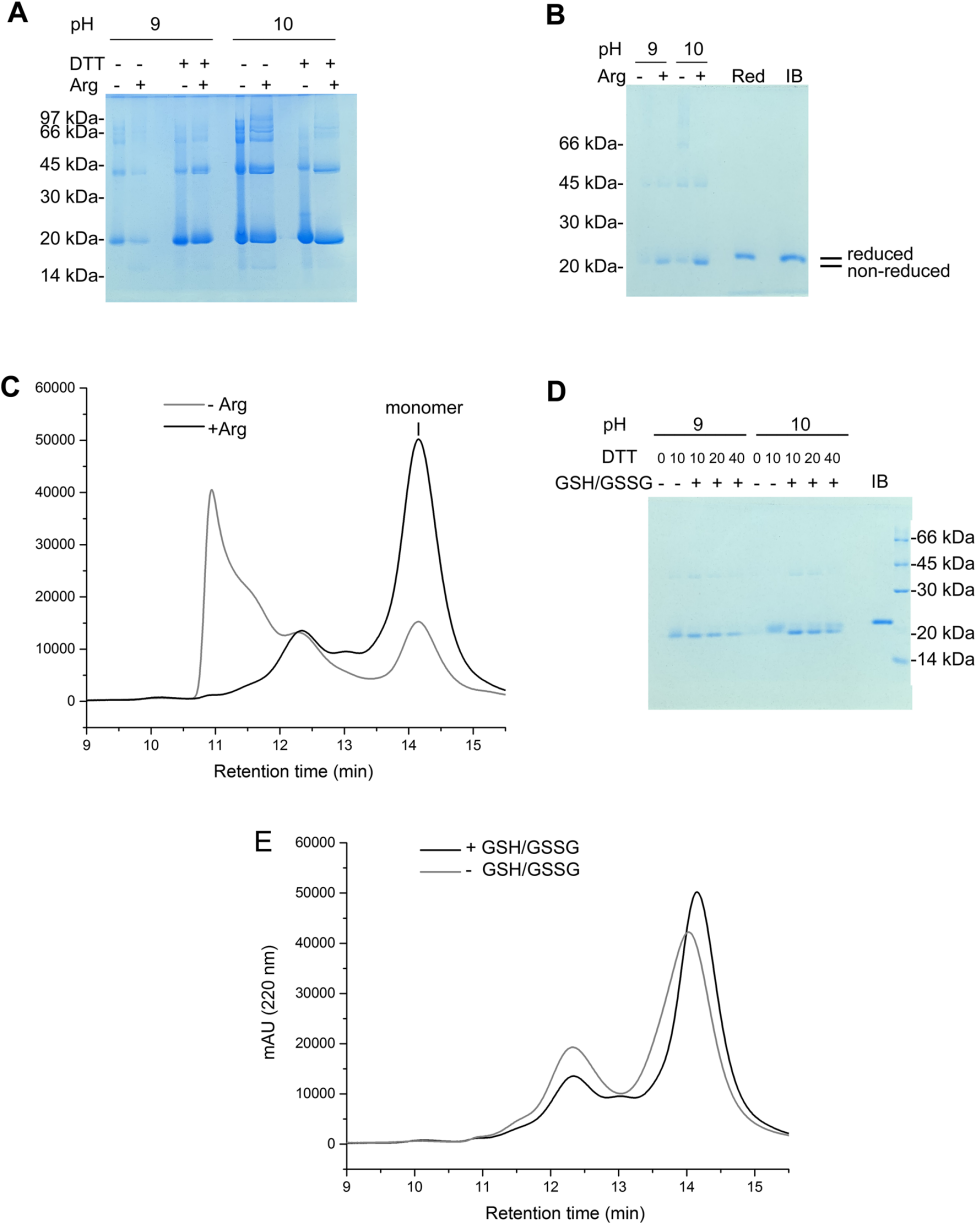
Dissociation of oligomers of hGH and oxidative folding. **A** SDS-PAGE of non-boiled and non-reduced supernatants of hGH-IBs suspensions at a concentration of 1 mg hGH/mL that were subjected to 2.4 kbar and pH 9.0 and 10.0 in the absence and in the presence of DTT (10 mM) and Arg (0.25 M); **B** SDS-PAGE of supernatants of hGH-IBs that were subjected to 2.4 kbar for 90 min at pH 9.0 and 10.0 in the presence of 10 mM DTT and in the presence and absence of Arg (0.25 M). For refolding, the supernatants of the centrifuged samples were diluted tenfold in Tris pH 7.4 in the presence of 2 mM GSSG, incubated and dialyzed. IB indicates the hGH-IBs, that was reduced and Red indicates a reduced soluble sample; **C** HPSEC analysis of the samples solubilized at pH 10.0 and treated as in **B**; **D** SDS-PAGE of samples refolded as in **B** in the presence of Arg and in the presence or in the absence of DTT and GSSG; E, HPSEC of samples solubilized at pH 10.0 and treated as in **D**

Most of the hGH solubilized at high pressure and pH 9.0 does not penetrate SDS-PAGE in the absence of reducing agent, but the intensity of hGH monomer band is enhanced when the protein was solubilized in the presence of DTT. hGH solubilized at pH 10.0 and 2.4 kbar also presents large quantities of high molecular weight species that produce a smear in SDS-PAGE, which is converted into mainly monomer and dimer when solubilized in the presence of DTT and Arg (Fig. 2A). These results indicate the presence of non-native intermolecular disulfide bonds in hGH, which are ruptured in the presence of DTT. The presence of Arg also improved oligomer dissociation.

Attempts were made to obtain monomeric hGH with correct C53–C165 and C182–C189 disulfide bonds from the protein solubilized at alkaline pH and high pressure. Considering that disulfide bond formation and shuffling can be promoted under the appropriate conditions using thiol-containing oxidized and reducing reagents [26], we chose to solubilize the protein in the presence of a reducing reagent and subsequently dilute the protein at physiological pH in the presence of a redox pair.

hGH solubilized in the absence of Arg forms high-order oligomers, while the presence of this amino acid during compression resulted in mostly monomeric protein (Fig. 2B). Consistent with these results, the percentage of the protein eluting as a monomer in HPSEC [peak with retention time (RT) of 14.1 min] improved from 22% when the protein was solubilized in the absence of Arg to 73% in the presence of Arg during compression, with a corresponding reduction in high molecular weight species with lower retention times (Fig. 2C).

The presence of reducing agents is also important for hGH refolding since, if no DTT is present during hGH-IBs solubilization, no monomeric or higher molecular weight bands are observed in SDS-PAGE. Increasing the concentration of DTT to greater than 10 mM, however, did not improve hGH refolding (Fig. 2D). The presence of the oxidizing reagent GSSG after solubilization is also an important factor for formation of hGH with correct disulfide bonds. hGH refolded in the absence of GSSG migrates slightly more slowly in SDS-PAGE and with lower retention time in HPSEC than the samples incubated in the presence of the reduction reagent, indicating that the protein is in a reduced or partially reduced state (Fig. 2D and E). We succeeded in obtaining correctly refolded protein when the solubilization was carried out in the presence of 10 mM DTT and 0.25 M Arg and the reduced protein was subsequently diluted 10 × in Tris HCl pH 7.4 in the presence of 2 mM oxidized glutathione (GSSG) followed by incubation at 4 °C for 48 h. The redox pair formed by 2 mM GSH (from 1 mM DTT that reduce 1 mM GSSG to 2 mM GSH) and 1 mM GSSG was able to form and shuffle the disulfide bonds, resulting in a good yield of predominantly monomers with intramolecular S–S bonds. It is important to note that the protein solubilized in the presence of 10 mM DTT and Arg and incubated in the presence of GSSG presents, as expected, a higher mobility in SDS-PAGE indicating a decreased protein volume occasioned by intramolecular S–S bonds than the hGH-IB that was reduced by boiling in the presence of 0.1 M DTT in the SDS-PAGE (Fig. 2D).

A refolding yield of 81% was obtained for hGH that was solubilized at pH 10.0. Approximately 73% of the protein is in a monomeric state (Fig. 2C). We found, therefore, an average yield of 413 mg refolded monomeric hGH/L culture. The refolding yield of hGH that was solubilized at pH 9.0 was lower: 58% with 60% monomer and, therefore, an average yield of 243 mg monomeric hGH was obtained from 1 L culture.

### Structure of refolded hGH

**Fig. 3:**
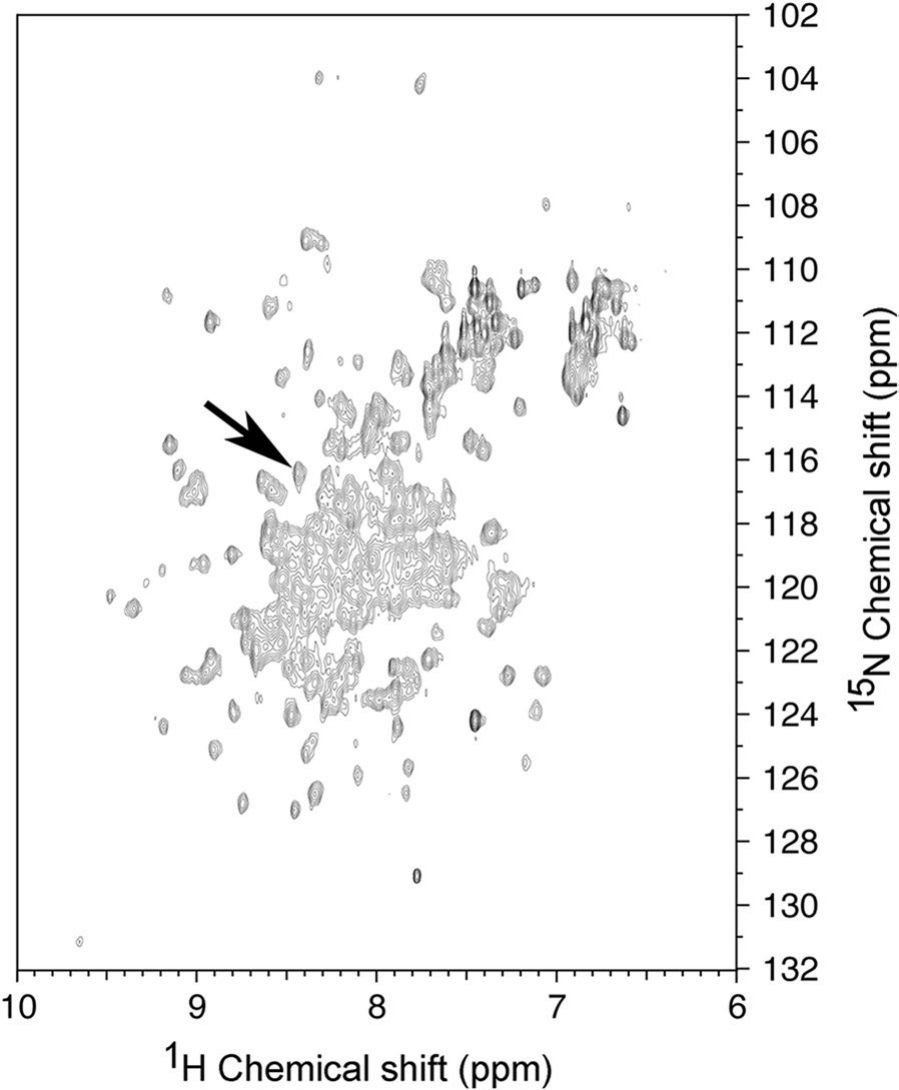
Refolded hGH is well folded and present expected disulfide bond. ^1^H–^15^N HSQC spectrum of hGH that was refolded and chromatographically separated from the higher molecular weight isoforms at pH 7.5. The arrow shows the expected position of the backbone amide crosspeak for residue C182 [27, 28]

In order to confirm that refolded hGH is structured and that the disulfide bonds formed are those encountered in the native protein, we expressed and purified ^15^N-labelled recombinant hGH for which the backbone amide ^15^N signals of two of the four cysteines (C53 and C182) had been previously assigned [27, 28]. Figure 3 shows the ^1^H–^15^N HSQC spectrum of hGH that was refolded and purified by SEC in the monomeric form. The C53 cross-peak is in a highly crowded region of the spectrum, but the cross-peak corresponding to C182 can be observed at the expected position (^15^N = 116.30 ppm and ^1^H = 8.42 ppm) (Fig. 3). This result indicates that the S–S bonds of refolded hGH are correct. Furthermore, the HSQC spectrum presents a large chemical shift dispersion in the ^1^H dimension that is characteristic of well-folded proteins [29].

#### Analysis of secondary and tertiary structure of hGH in IBs

**Fig. 4:**
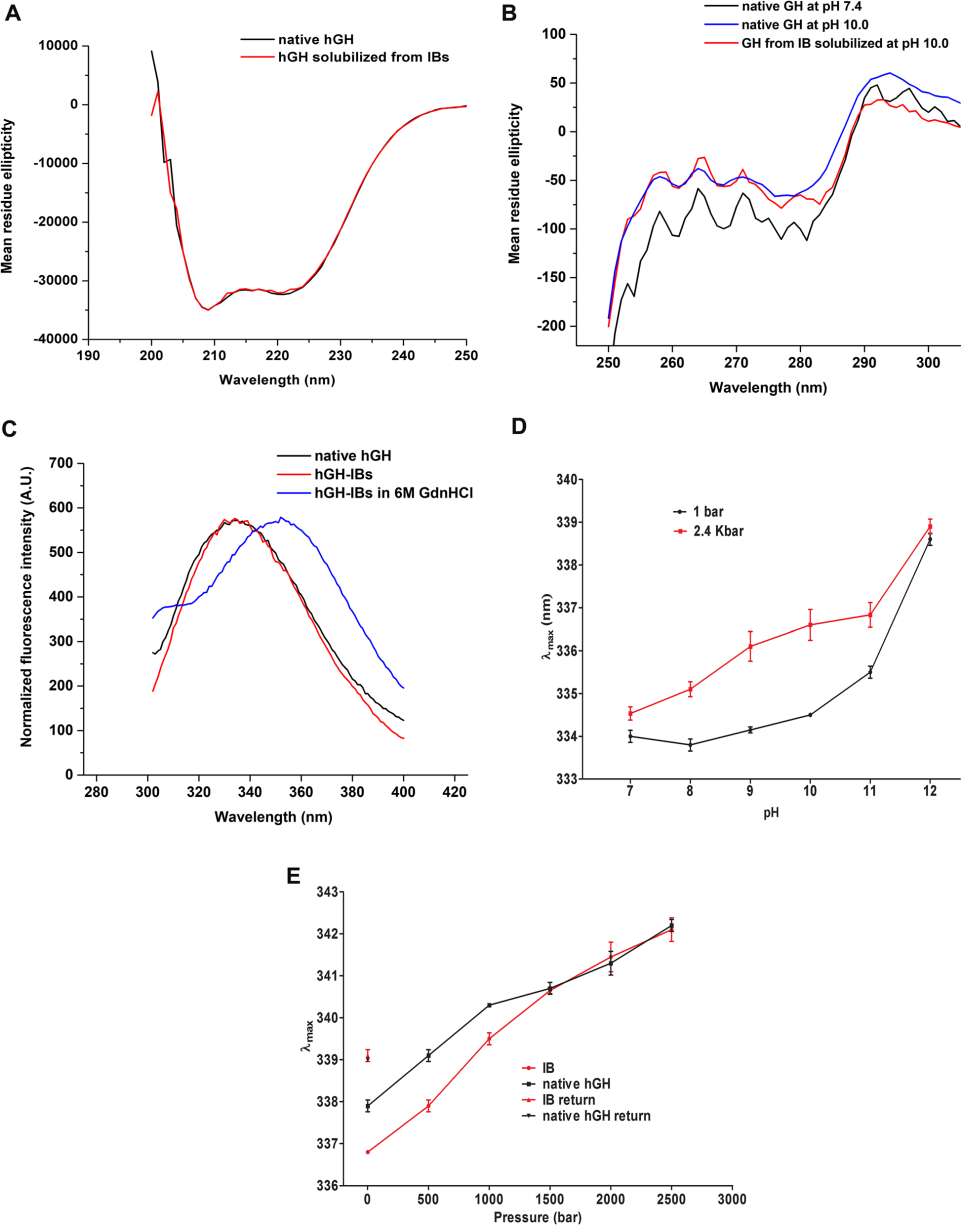
Analysis of secondary and tertiary structure of hGH in hGH-IBs. **A** Far-UV CD spectra of native hGH and of hGH that had been solubilized from hGH-IBs at pH 10.0 and 2.4 kbar; **B** near-UV CD spectra of native hGH at pH 7.4, at pH 10.0 and of hGH that was solubilized from hGH-IBs at pH 10.0 and 2.4 kbar; **C** fluorescence spectra of native hGH, of hGH-IBs and of hGH-IBs that was denatured by incubation in 6 M GdnHCl; **D** fluorescence λ_max_ of supernatants of hGH-IBs that were subjected to 2.4 kbar or at 1 bar in pH from 7.0 to 12.0; **E** fluorescence λ_max_ of refolded hGH and of hGH-IBs, both at pH 10.0 with 10 mM DTT and 0.25 M Arg subjected to increasing pressure levels

Structural analysis of hGH in the IBs was facilitated by its high purity and the fact that the two parameters that improve solubilization, pH 10.0 and high pressure, do not interfere with the CD analysis. hGH is an all α-helical protein with five α-helices and 52% α-helical content. The near-UV CD spectrum of native hGH was compared to the protein that was solubilized from IBs at 2.4 kbar and pH 10.0. The two spectra are indistinguishable and both present residual ellipticity minima at 208 and 222 nm characteristic of α-helical proteins (Fig. 4A) and which has been previously reported for native hGH [30]. Therefore, by this criterium, hGH in IBs presents a native-like secondary structure.

The near-UV CD spectra of hGH features a positive band with a maximum at 292 nm, that is assigned to the tryptophan, a negative band centered at 278 nm assigned to tyrosine and a negative doublet with minima at 260 and 267 nm assigned to phenylalanines [31]. The near-UV CD spectra of native hGH at pH 7.4 presents these general features. A shift of the bands at 278, 260 and 267 nm to fewer lower values was observed upon increasing alkalinity to pH 10.0. Also, the spectrum of hGH that was solubilized from hGH-IBs at pH 10.0 and 2.4 kbar is very similar to the spectrum of native hGH incubated at this pH (Fig. 4B). This result shows elevated pH was the factor that caused the spectral shifts of both the native hGH and the protein in hGH-IBs, indicating that the protein in hGH-IBs has a native-like tertiary structure.

hGH contains eight Tyr and one Trp residues. In the folded state, Tyr residue fluorescence emission is suppressed due to energy transfer to Trp. The Trp emission peak has a maximum at 335 nm, indicating that this residue is partially buried in the hydrophobic core of the protein. In the denatured state (6 M GdnHCl), Tyr residues are separated from Trp, energy transfer is suppressed and Tyr emission at 305 nm can be observed while the Trp emission maximum is red-shifted to 352 nm [5, 31]. As expected, fluorescence peaks at 305 and at 352 nm are observed in hGH-IBs denatured in the presence of 6 M GdnHCl. The fluorescence of native hGH at pH 7.4 present a single peak with maximum emission at 334 nm, which indicates that Trp residue are retained in the hydrophobic core, that the Tyr are in its vicinity and that the protein adopts a native tertiary structure. A similar fluorescence profile (λ max = 334 nm) is observed for hGH-IBs (Fig. 4C).

The fluorescence of suspensions of hGH-IBs that had been subjected to 2.4 kbar and pH varying from 7 to 12 were determined in order to verify if hGH native-like tertiary structure is maintained during application of the conditions used for solubilization. The λ_max_ of hGH-IBs incubated at 1 bar and pH 7.0 is 334.0 ± 0.1 and red-shifts were observed at increased pressure and pH. The λ_max_ of hGH subjected to 2.4 kbar at pH 9.0 and 10.0 were respectively 336.1 ± 0.4 and 336.8 ± 0.4 with single peaks that were 2.1 and 2.8 nm red-shifted in relation to the native protein, but 15.2 nm blue-shifted in relation to the completely denatured protein (352 nm) (Fig. 4D). These results indicate that the conditions we used for solubilization were mild and did not unfold the protein.

In order to determine if insoluble and solubilized hGH from the IBs behave similarly to the native protein, hGH-IBs were suspended in the buffer utilized for solubilization and refolding (pH 10.0, 10 mM DTT, 0.25 M Arg and 1 mM EDTA) under increasing pressures. Fluorescence was monitored during compression and the data were compared to those obtained for the native protein. Surprisingly, the λ_max_ of hGH-IBs at atmospheric pressure presented a slightly lower value (336.8 ± 0.0) than the one obtained for native hGH (337.9 ± 0.1) and this difference was maintained up to 1.0 kbar. At pressures of 1.5 kbar and above, similar values of λ_max_ were obtained for both proteins reaching the maximum value of 342.2 ± 0.2 at 2.5 kbar with a single peak (Fig. 4E). We suggest that the protein in hGH-IBs under these conditions can be partially protected, but when it is solubilized at 1.5 kbar, its structure is similar to that of the native protein. The λ_max_ presents a blue-shift after decompression to 339.0 nm and this value was also very similar for both treatments. The results reinforce the idea of native-like tertiary structure in hGH-IBs.

## Discussion

The solubilization of human growth hormone IBs (hGH-IBs) under an extremely mild, non-denaturing condition, i.e. the association of alkaline pH and high pressure, served two purposes in this study: the determination of protein structure in IBs, by analysis of the solubilized protein and the establishment of a refolding protocol. We determined that the protein obtained in this manner presents a profile similar to the native one at the same condition and, furthermore, that hGH in IBs presents native-like secondary and tertiary structures.

Biological activity, rather than the determination of tertiary structure, is generally used to analyze proteins within IBs [15, 32, 33]. We have shown in our study that Trp intrinsic fluorescence is a very simple, sensitive and efficient method to analyze proteins in IBs. We observed that hGH-IBs fluorescence presents the same λ_max_ as the native protein, indicative of a native-like tertiary structure. Solubilization of the IBs using the mild conditions described maintains native-like structure and minimizes reaggregation.

Alkaline pH is an interesting condition for solubilization of aggregates, induced by electrostatic repulsion. Extremely alkaline pH, however, can provoke protein unfolding, as shown by studies using bovine serum albumin (BSA). Neither secondary nor tertiary contacts of BSA are lost upon incubation at pH 9.0. At higher pH, however, tertiary structure is disrupted and a BSA molten globule is formed [19, 34]. Molten globules are compact polypeptide chains with substantial secondary structure, but no specific tertiary structure with most nonpolar residues exposed to the solvent [35]. This state should be avoided during solubilization prior to refolding because it is usually prone to aggregation. Besides, chemical modifications of proteins can occur at very alkaline pH [36]. Our study shows that the concomitant use of high pressure diminished the pH necessary to efficiently solubilize hGH-IBs, thereby reducing the possibility of re-aggregation and of chemical modifications.

Previous reports have employed combinations of 30% trifluoroethanol and 3 M urea or 6 M n-propanol in the presence of 2 M urea, described as mild, to solubilize hGH-IBs prior to subsequent refolding. While hGH secondary structure was maintained, native tertiary structure is completely lost in both conditions [5, 37].

Our ability in this study to solubilize hGH without losing tertiary structure, as indicated by the very small shift in Trp fluorescence λ maximum (Fig. 4), is likely responsible for the very small amount of protein aggregation and high yield of refolded protein obtained.

## Conclusions

We showed that native-like secondary and tertiary structure are present in hGH-IBs, opening the way to new perspectives for different types of applications. The refolding process could be extended with high yields to a wide range of heterologously expressed recombinant proteins, with a great potential for application in research and in industry.

## Data Availability

All data generated or analysed during this study are included in this published article.

## Abbreviations

DTT: Dithiothreitol
EDTA: Ethylenediaminetetraacetic acid
GdnHCl: Guanidine hydrochloride
GSH: Reduced glutathione
GSSG: Oxidized glutathione
hGH-IBs: Inclusion bodies of hGH
IBs: Inclusion bodies
IPTG: Isopropyl b-d-thiogalactopyranoside
LS: Light scattering
SDS-PAGE: Sodium dodecyl sulphate polyacrylamide gel electrophoresis
